# Changes in alveolar bone support induced by the Herbst appliance: a tomographic evaluation

**DOI:** 10.1590/2177-6709.21.2.095-101.oar

**Published:** 2016

**Authors:** João Paulo Schwartz, Taisa Boamorte Raveli, Humberto Osvaldo Schwartz-Filho, Dirceu Barnabé Raveli

**Affiliations:** 1 PhD resident, Universidade Estadual Paulista (UNESP), Department of Orthodontics, Araraquara, São Paulo, Brazil.; 2 Adjunct Professor, Universidade Federal do Paraná (UFPR), Department of Stomatology, Curitiba, Paraná, Brazil.; 3 Professor, Universidade Estadual Paulista (UNESP), Department of Orthodontics, Araraquara, São Paulo, Brazil.

**Keywords:** Periodontium, Activator appliances, Cone-beam computed tomography

## Abstract

**Objective:**

This study evaluated alveolar bone loss around mandibular incisors, induced by the Herbst appliance.

**Methods:**

The sample consisted of 23 patients (11 men, 12 women; mean age of 15.76 ± 1.75 years), Class II, Division 1 malocclusion, treated with the Herbst appliance. CBCT scans were obtained before treatment (T_0_) and after Herbst treatment (T_1_). Vertical alveolar bone level and alveolar bone thickness of mandibular incisors were assessed. Buccal (B), lingual (L) and total (T) bone thicknesses were assessed at crestal (1), midroot (2) and apical (3) levels of mandibular incisors. Student's t-test and Wilcoxon t-test were used to compare dependent samples in parametric and nonparametric cases, respectively. Pearson's and Spearman's rank correlation analyses were performed to determine the relationship of changes in alveolar bone thickness. Results were considered at a significance level of 5%.

**Results:**

Mandibular incisors showed no statistical significance for vertical alveolar bone level. Alveolar bone thickness of mandibular incisors significantly reduced after treatment at B1, B2, B3, T1 and significantly increased at L2. The magnitude of the statistically significant changes was less than 0.2 mm. The changes in alveolar bone thickness showed no statistical significance with incisor inclination degree.

**Conclusions:**

CBCT scans showed an association between the Herbst appliance and alveolar bone loss on the buccal surface of mandibular incisors; however, without clinical significance.

## RESUMO

**Introdução:**

este estudo avaliou a perda óssea alveolar ao redor dos incisivos inferiores induzida pelo aparelho de Herbst.

**Métodos:**

a amostra foi composta por 23 pacientes (11 homens e 12 mulheres; média de idade 15,76 ± 1,75 anos), má oclusão de Classe II, divisão 1, tratados com aparelho de Herbst. TCFCs foram realizadas antes do tratamento (T_0_) e após o tratamento (T_1_) com o Herbst. A altura e a espessura óssea alveolar dos incisivos inferiores foram avaliadas. As espessuras ósseas vestibular (V), lingual (L) e total (T) foram mensuradas nos terços cervical (1), médio (2) e apical (3) dos incisivos inferiores. O teste *t* de Student e o teste *t* de Wilcoxon compararam as amostras dependentes nos casos paramétricos e não paramétricos, respectivamente. As análises de Pearson e Spearman determinaram a correlação entre as alterações na espessura do osso alveolar. Os resultados foram considerados para um nível de significância de 5%.

**Resultados:**

os incisivos inferiores não apresentaram significância estatística para a altura óssea alveolar. Após o tratamento, a espessura óssea alveolar dos incisivos inferiores reduziu-se significativamente em V1, V2, V3 e T1 e aumentou significativamente em L2. A quantidade da alteração óssea significativa foi menor que 0,2mm. As alterações na espessura óssea alveolar não apresentam correlação estatisticamente significativa com o grau de inclinação do incisivo.

**Conclusões:**

as imagens de TCFC demonstram associação entre o uso do aparelho de Herbst e a perda óssea alveolar no lado vestibular dos incisivos inferiores; entretanto, sem significância clínica.

## INTRODUCTION

Angle Class II relationship is the malocclusion most commonly found in the orthodontic practice;^1^ approximately one third of all patients present Class II, Division 1 malocclusion,^2^ and mandibular deficiency is the primary etiological factor.^2^

Clinical practice and researches have shown that the Herbst appliance is effective in correcting Class II malocclusion.^3^ ^,^ ^4^ The Herbst appliance is a fixed functional appliance that induces dentoalveolar changes and buccal movement of mandibular incisors.^5^ ^-^ ^11^

Compensatory orthodontic treatment of Class II malocclusion requires mandibular incisors to be proclined. Due to this fact, alveolar bone around incisors should be considered. The presence of harmful habits can alter the periodontal status and, in association with proclined mandibular incisors, could result in gingival recession.^12^ ^,^ ^13^

Evaluation of orthodontic treatment effects produced by the Herbst appliance has been performed by periapical, panoramic and cephalometric radiographs. Buccal and lingual alveolar bone plates are not correctly visualized in two-dimensional radiographs due to overlapping images. Cone-beam computed tomograph (CBCT) scans allow evaluation of periodontal tissue support tridimensionally. Researchers have been recently studying alveolar bone changes induced by orthodontic tooth movement with different voxel sizes.^14^ ^-^ ^17^

Knowledge of changes in periodontal tissue support induced by tooth movement is important, and there are no studies in the literature relating alveolar bone changes induced by the Herbst appliance by means of CBCT scans.

This research aimed at evaluating alveolar bone changes around mandibular incisors, induced by orthodontic treatment with the Herbst appliance. 

## MATERIAL AND METHODS

This retrospective study was reviewed and approved by the Ethics Committee of Universidade Estadual Paulista (FOAr-UNESP), School of Dentistry, Araraquara, São Paulo, Brazil. Patients were selected in local public schools. A total of 30 patients who presented skeletal Class II, Division 1 malocclusion were invited to participate in the study, following the inclusion criteria. Five patients refused to participate and two left the study before its conclusion. A total of 23 patients (11 men, 12 women; mean age of 15.76 ± 1.75 years) were sequentially treated by an orthodontist at the Department of Universidade Estadual Paulista (FOAr-UNESP), School of Dentistry, Araraquara, São Paulo, Brazil.

Skeletal Class II, Division 1 malocclusion was diagnosed by facial and occlusal analyses. Inclusion criteria were: convex profile; straight nasolabial angle; short mentocervical line; molar and canines in bilateral Class II relationship, equal or higher than the half of a cusp; overjet equal or greater than 5 mm; absence of posterior crossbite; absence of dental crowding; and complete permanent dentition, except third molars.^18^ Exclusion criteria were: syndromic patient, extreme vertical growth pattern and prior orthodontic treatment.^18^

Patients used banded Herbst appliance until eight months of treatment were completed (mean 8.50 ± 0.70 months), with single mandibular advancement until incisors were in an edge-to-edge relationship.^8^ ^,^ ^18^ The telescopic mechanism used was the Flip-Lock Herbst^TM^ (TP Orthodontics, Inc.) model constituted by connectors, tubes and pistons.

A transpalatal fixed bar was used for upper anchorage, secured to first molars. The bar was made of 1.2-mm steel wire, 2 mm distant from the palate and with an extension of 1.2-mm steel wire to the second molar.^18^ In the lower arch, a Nance lingual arch modified for the Herbst appliance was attached to first molars. It was made of 1.2-mm steel wire and located 3 mm distant from incisors lingual face. Anchorage appliances were constructed by the same technician.^18^

To evaluate alveolar bone loss around mandibular incisors, induced by the Herbst appliance, CBCT scans were obtained before treatment (T_0_) and after treatment (T_1_). Patients were scanned in an upright position with maximum intercuspation. To this end, i-CAT^TM^ Classic (Imaging Sciences International, Hatfield, PA, USA) was used, with a 17 x 13.3 cm field of view, 120 kVp tube voltage, 18.45 mA tube current and 0.4 mm isometric voxel. CBCT scans were examined by means of Dolphin^TM^ Imaging software (Dolphin Imaging and Management Solutions, Chatsworth, Calif., USA) by means of multiplanar reconstruction (axial, sagittal and coronal) and two-dimensional reconstruction of lateral cephalogram.

**Figure 1:**
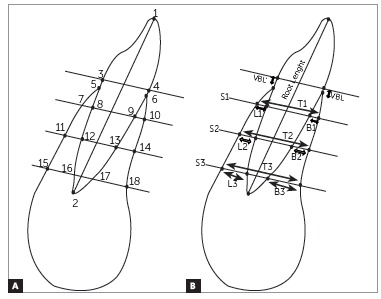
Reference points (A) and measurements (B) used to evaluate alveolar bone height and thickness.

**Figure 2:**
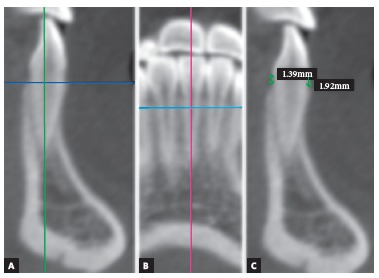
Measurements used to evaluate alveolar bone height. Sagittal multiplanar reconstruction, coronal cursor adjusted in tooth long axis (A). Coronal multiplanar reconstruction, sagittal cursor adjusted in tooth long axis (B). Buccal and lingual alveolar bone height (C).

Tables 1 and 2 show reference points and measurements used to evaluate alveolar bone height and thickness (Fig 1). The coronal and sagittal cursor was adjusted in the tooth long axis (incisal edge center to root apex), according to the tooth of interest ^19^ (Fig 2). Buccal and lingual alveolar bone heights were evaluated in sagittal multiplanar reconstruction. Measurement was taken from the most superior point of crestal alveolar bone to the cemento-enamel junction (CEJ), being a parallel line to the tooth long axis^14^ (Fig 2).

**Table 1:** Reference points and definitions used to evaluate alveolar bone height and thickness.

Points	Definitions
1	Incisal edge
2	Root apex
3	Lingual CEJ
4	Buccal CEJ
5	Lingual alveolar crest
6	Buccal alveolar crest
7	Lingual symphysis crestal level
8	Lingual root crestal level
9	Buccal root crestal level
10	Buccal symphysis crestal level
11	Lingual symphysis midroot
12	Lingual root midroot level
13	Buccal root midroot level
14	Buccal symphysis midroot level
15	Lingual symphysis apical level
16	Lingual root apical level
17	Buccal root apical level
18	Buccal symphysis apical level

**Table 2:** Definitions of measurements used to evaluate alveolar bone height and thickness.

Measurements	Definitions
Vertical bone lingual (VBL')	Distance between points 3 and 5
Vertical bone buccal (VBL)	Distance between points 4 and 6
Lingual bone crestal level (L1)	Distance between points 7 and 8
Buccal bone crestal level (B1)	Distance between points 9 and 10
Total bone crestal level (T1)	Distance between points 7 and 10
Lingual bone midroot level (L2)	Distance between points 11 and 12
Buccal bone midroot level (B2)	Distance between points 13 and 14
Total bone midroot level (T2)	Distance between points 11 and 14
Lingual bone apical level (L3)	Distance between points 15 and 16
Buccal bone apical level (B3)	Distance between points 17 and 18
Total bone apical level (T3)	Distance between points 15 and 18
Long Axis	Distance between points 1 and 2

**Figure 3:**
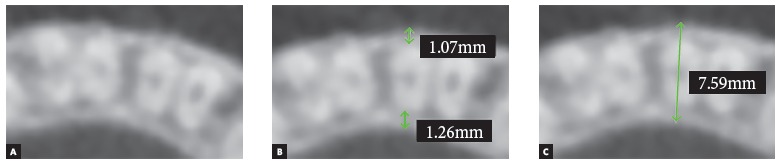
Measurements used to evaluate alveolar bone thicknesses. Axial multiplanar reconstruction (A). Buccal and lingual bone thickness (B). Total bone thickness.

Buccal (V), lingual (L) and total (T) bone thicknesses were assessed in each tooth by axial multiplanar reconstruction in three levels.^17^ Axial slices were 3 mm distant from each other, and so was the reference point CEJ, being the three slices established at sagittal multiplanar reconstruction parallel to CEJ (Fig 1). The most buccal and lingual points were established at the alveolar bone plate and tooth root to measure buccal bone thickness (buccal bone point to buccal tooth root point), lingual bone thickness (lingual bone point to lingual tooth root point) and total bone thickness (buccal bone point to lingual bone point) in the three axial levels (Fig 3).

Measurements were reevaluated randomly after two weeks by the same blinded examiner. The error of the method was evaluated by Intraclass Correlation Coefficient (ICC). Shapiro-Wilk test was used to assess normal distribution, and Student's t-test as well as Wilcoxon t-test were used to compare dependent samples in parametric and nonparametric cases, respectively. Pearson's and Spearman's rank correlation analyses were performed to determine the relationship of changes in alveolar bone thickness. Results were considered at a significance level of 5%. Statistical analysis was performed by means of SPSS^TM^ (SPSS Inc, Chicago, III) and GraphPad Prism^TM^ (GraphPad Prism Inc, San Diego, USA).

## RESULTS

Systematic intraexaminer error indicated excellent reliability (ICC = 0.91). Table 3 shows the means and standard deviations for cephalometric measurements at T_0_ and T_1_ for all subjects. Significant differences were found in SNB, ANB, WITS and IMPA measurements, showing the changes induced by the Herbst appliance. Table 4 shows means and standard deviations of changes in alveolar bone around mandibular incisors at T_0_ and T_1_. There were no statistical differences for buccal and lingual vertical alveolar bone level of mandibular incisors during treatment.

**Table 3:** Mean, standard deviation (SD) and level of significance (*p*) of cephalometrics measures.

Measurements	T_0_ (Mean ± SD)	T_1_ (Mean ± SD)	*p* value
SNA (degrees)	81.69 ± 4.11	81.62 ± 3.81	0.836
SNB (degrees)	77.66 ± 3.88	78.49 ± 3.66	0.027*
ANB (degrees)	4.34 ± 2.16	3.47 ± 2.17	0.000**
WITS (mm)	4.49 ± 2.76	3.47 ± 2.72	0.010*
IMPA (degrees)	98.39 ± 7.00	103.00 ± 7.90	0.000**
1.1 (degrees)	116.60 ± 9.99	116.90 ± 9.07	0.805

*p <0.05; **p <0.001.

**Table 4:** Mean, standard deviation (SD) and level of significance (P) of alveolar bone height and thickness in the lower incisors.

Measurements	T_0_ (Mean ± SD)	T_1_ (Mean ± SD)	T_1_-T_0_ (Mean ± SD)	*p* value
Buccal height (VBL) (mm)	1.41 ± 0.43	1.54 ± 0.53	0.13 ± 0.07	0.090
Lingual height (VBL') (mm)	1.43 ± 0.50	1.52 ± 0.50	0.09 ± 0.00	0.132
Lingual crestal (L1) (mm)	0.76 ± 0.40	0.70 ± 0.42	-0.06 ± 0.01	0.300
Buccal crestal (B1) (mm)	0.60 ± 0.26	0.44 ± 0.25	-0.16 ± 0.00	0.000***
Total crestal (T1) (mm)	7.03 ± 0.73	6.90 ± 0.74	-0.13 ± 0.00	0.010*
Lingual midroot (L2) (mm)	1.16 ± 0.52	1.36 ± 0.65	0.20 ± 0.09	0.000***
Buccal midroot (B2) (mm)	0.78 ± 0.42	0.60 ± 0.40	-0.18 ± 0.01	0.000***
Total midroot (T2) (mm)	7.06 ± 0.92	7.08 ± 0.96	0.02 ± 0.02	0.862
Lingual apical (L3) (mm)	1.85 ± 0.87	1.98 ± 0.86	0.13 ± 0.00	0.078
Buccal apical (B3) (mm)	1.98 ± 0.93	1.84 ± 0.87	-0.14 ± 0.04	0.035*
Total apical (T3) (mm)	7.66 ± 1.35	7.69 ± 1.35	0.03 ± 0.00	0.705

*p < 0.05; ***p < 0.001.

There was statistical significant difference for buccal and total alveolar bone thickness at the crestal level, showing a reduction of mean values from T_0_ to T_1_. Alveolar bone thickness at the midroot level showed statistical significant difference for lingual and buccal surfaces, with an increase and reduction of means during treatment, respectively. Mean alveolar bone thickness at the apical level decreased, showing a significant difference from T_0_ to T_1_ (Table 4). Alveolar bone thickness increased at the midroot level and reduced at the crestal level, midroot and apical levels for lingual and buccal sides, respectively. 

The magnitude of statistically significant changes for alveolar bone thickness was less than 0.2 mm (Table 4). There was no statistically significant correlation between incisor inclination degree and extension of changes in alveolar bone thickness around mandibular incisors (Table 5).

**Table 5:** Pearson's and Spearman's rank correlation analysis between mandibular incisors inclination and alveolar bone changes.

Variable	Pearson's correlation		Spearman's correlation	
	Coefficient	p value	Coefficient	p value
Buccal height (VBL) (mm)	0.209	0.337	0.208	0.339
Lingual height (VBL') (mm)	0.401	0.057	0.272	0.208
Lingual crestal (L1) (mm)	0.143	0.514	0.051	0.815
Buccal crestal (B1) (mm)	-0.314	0.143	-0.248	0.253
Total crestal (T1) (mm)	-0.085	0.698	-0.098	0.653
Lingual midroot (L2) (mm)	0.409	0.052	0.385	0.069
Buccal midroot (B2) (mm)	-0.157	0.474	0.036	0.868
Total midroot (T2) (mm)	0.141	0.519	0.226	0.297
Lingual apical (L3) (mm)	-0.005	0.980	-0.227	0.296
Buccal apical (B3) (mm)	0.168	0.441	0.189	0.385
Total apical (T3) (mm)	0.313	0.145	0.360	0.360

## DISCUSSION

This CBCT study evaluated alveolar bone loss around mandibular incisors, induced by the Herbst appliance. Patients with a mean age of 15.76 years comprised the group to simulate the postpubertal period, a stage during which Class II treatment with the Herbst appliance shows more dentoalveolar than skeletal response.^4^ Cephalometric measurements SNB, ANB, WITS and IMPA showed significant statistical differences (Table 3), confirming appliance effectiveness and changes induced by the mechanic of mandibular advancement during correction of skeletal Class II malocclusion. These results are similar to related articles in the literature.^5^ ^-^ ^11^

Alveolar bone support is essential to teeth stability and periodontal health. Optimal stability of mandibular incisors is considered when the tooth is positioned in the medullary portion of the alveolar bone and it is found in good balance with labial and lingual musculature.^20^ The mandibular symphysis is an anatomical structure that limits the buccal and lingual movement of incisors, shows thin alveolar bone plate and is susceptible to periodontal disease.^21^ Previous studies have shown that excessive inclination of incisors buccally or lingually must be avoided, thereby preventing alveolar bone loss and consequent loss of tooth bone support.^22^ ^,^ ^23^ ^,^ ^24^ This shows the importance of our study because there is no literature evaluating the effect of forward movement of mandibular incisors induced by the Herbst appliance in alveolar bone tridimensionally.

Lingual alveolar bone thickness presented statistically significant difference and increased at the midroot level (Table 3). Buccal bone thickness presented statistically significant difference and reduced at the crestal, midroot and apical levels (Table 3). Even with the use of anchorage with a lingual arch modified for the Herbst appliance, distant from incisors lingual surface, and a transpalatal fixed bar at the upper arch, mandibular incisors proclined significantly. There was a statistically significant decrease in total bone thickness at the crestal level (Table 3). Changes in total bone thickness are related to changes in inclination and intrusion extension of mandibular incisors.^17^ ^,^ ^25^ As previously mentioned, there is no literature that reports assessing alveolar bone thickness induced by the Herbst appliance by means of CBCT scans; therefore, there are no parameters for comparison of our results. 

Alveolar bone thickness with statistically significant changes was less than 0.2 mm, and this result is similar to that achieved by Lee et al^14^ who evaluated alveolar bone loss around mandibular incisors with similar protocols of tomographic image acquisition. A limitation of this study could be that the magnitude of statistically significant changes is smaller than the voxel size. However, Yodthong, et al^17^ evaluated alveolar bone thickness during maxillary incisors retraction with 0.125-mm voxel resolution, and found mean alveolar bone changes similar to our study. Moreover, the mean alveolar bone thickness and vertical level are larger than the voxel size, similarly to Kook et al^13^ and Lee et al.^14^ One of the discussions regarding tomographic image acquisition for evaluation of alveolar bone is voxel size. Tomographic image accuracy to measure bone thickness around mandibular anterior teeth under different resolutions showed no significant statistical difference between voxel protocols.^26^ Despite statistically significant alveolar bone changes induced by the Herbst appliance, the minimal thickness reduction at the buccal surface of mandibular incisors has no clinical significance in patients in good periodontal health and without harmful habits. 

Orthodontic proclination of mandibular incisors by the Herbst appliance does not result in gingival recession.^27^ There is no association between buccal movement of mandibular incisors and the occurrence of gingival recession.^12^ The periodontal status must be evaluated regarding health, the amount of keratinized gingiva, mucogingival problems and harmful habits, such as smoking.^28^ The association between these periodontal conditions pre- or postorthodontic treatment, with proclination of mandibular incisors, could result in gingival recession. 

There was no statistical difference between the inclination degree of mandibular incisors and changes in alveolar bone (Table 5). Alveolar bone change is related to biomechanical phenomena and is influenced by many factors, including periodontal environment, gingival type and oral habit of patient.^29^ Thus, it might be possible that the extent of alveolar bone change is not mathematically or directly correlated with the degree of incisor inclination.

Regarding tomographic image acquisition, the accuracy of CBCT scans under different voxel resolutions (0.125 and 0.4 mm) for linear measurement of alveolar bone thickness around mandibular incisors was evaluated and there was no significant statistical difference between these voxel protocols.^26^ However, when alveolar bone thickness is larger than the voxel size (0.4 mm), measurements are susceptible to be overestimated, and when it is close or smaller than the voxel size, it tends to be underestimated.^30^ Alveolar bone changes smaller than the voxel size could be a limitation of our study. 

In spite of the clinical relevance of the present results, we cannot underestimate that this is a retrospective study with methodological limitations. Therefore, further prospective studies must be performed with a larger sample size, including a control group, tomographic image acquisition, protocols (smaller voxel size, smaller field of view, higher spatial resolution and smaller noise from scatter) and long-term evaluations of alveolar bone remodeling after the end of treatment.

## CONCLUSION

Tridimensional evaluation by means of CBCT scans revealed an association between the Herbst appliance and alveolar bone loss at the buccal surface of mandibular incisors; however, thickness of bone changes was minimal and clinically irrelevant.

## References

[1] Ast DB, Carlos JP, Cons NC (1965). The prevalence and characteristics of malocclusion among Senior High School Students in Upstate New York. Am J Orthod.

[2] Jr. McNamara JA (1981). Components of Class II malocclusion in children 8-10 years of age.. Angle Orthod..

[3] Pancherz H (2000). Dentofacial orthopedics or orthognathic surgery is it a matter of age?. Am J Orthod Dentofacial Orthop.

[4] Ruf S, Pancherz H (2003). When is the ideal period for Herbst therapy-early or late. Semin Orthod.

[5] Barnett GA, Higgins DW, Major PW, Flores-Mir C (2008). Immediate skeletal and dentoalveolar effects of the crown-or banded type Herbst appliance on Class II division 1 malocclusion. Angle Orthod.

[6] El-Fateh T, Ruf S. (2011). Herbst treatment with mandibular cast splints: revisited. Herbst treatment with mandibular cast splints--revisited.. Angle Orthod..

[7] Obijou C, Pancherz H. (1997). Herbst appliance treatment of Class II, division 2 malocclusions.. Am J Orthod Dentofacial Orthop..

[8] Pancherz H (1982). The mechanism of Class II correction in Herbst appliance treatment A cephalometric investigation. Am J Orthod.

[9] Pancherz H (1979). Treatment of class II malocclusions by jumping the bite with the Herbst appliance A cephalometric investigation. Am J Orthod.

[10] von Bremen J, Pancherz H, Ruf S (2007). Reduced mandibular cast splints an alternative in Herbst therapy A prospective multicentre study. Eur J Orthod.

[11] Weschler D, Pancherz H (2005). Efficiency of three mandibular anchorage forms in Herbst treatment a cephalometric investigation. Angle Orthod.

[12] Kalha A (2013). Gingival recession and labial movement of lower incisors. Evid Based Dent.

[13] Kook YA, Kim G, Kim Y. (2012). Comparison of alveolar bone loss around incisors in normal occlusion samples and surgical skeletal class III patients.. Angle Orthod..

[14] Lee KM, Kim YI, Park SB, Son WS. (2012). Alveolar bone loss around lower incisors during surgical orthodontic treatment in mandibular prognathism.. Angle Orthod..

[15] Lund H, Gröndahl K, Gröndahl HG (2010). Cone beam computed tomography for assessment of root length and marginal bone level during orthodontic treatment. Angle Orthod.

[16] Lund H, Gröndahl K, Gröndahl HG (2012). Cone beam computed tomography evaluations of marginal alveolar bone before and after orthodontic treatment combined with premolar extractions. Eur J Oral Sci.

[17] Yodthong N, Charoemratrote C, Leethanakul C (2013). Factors related to alveolar bone thickness during upper incisor retraction. Angle Orthod.

[18] Schwartz JP, Raveli TB, Almeida KCM, Schwartz-Filho HO, Raveli DB (2015). Cone Beam Tomography study of apical root resorption induced by Hebst Appliance. J Appl Oral Sci.

[19] Timock AM, Cook V, McDonald T, Leo MC, Crowe J, Benninger BL (2011). Accuracy and reliability of buccal bone height and thickness measurements from cone-beam computed tomography imaging. Am J Orthod Dentofacial Orthop.

[20] Sarikaya S, Haydar B, Ciger S, Ariyürek M. (2002). Changes in alveolar bone thickness due to retraction of anterior teeth.. Am J Orthod Dentofacial Orthop..

[21] Yamada C, Kitai N, Kakimoto N, Murakami S, Furukawa S, Takada K. (2007). Spatial relationships between the mandibular central incisor and associated alveolar bone in adults with mandibular prognathism.. Angle Orthod..

[22] Ten Hoeve A, Mulie RM (1976). The effect of antero-postero incisor repositioning on the palatal cortex as studied with laminagraphy. J Clin Orthod.

[23] Vardimon AD, Oren E, Ben-Bassat Y (1998). Cortical bone remodeling/tooth movement ratio during maxillary incisor retraction with tip versus torque movements. Am J Orthod Dentofacial Orthop.

[24] Wainwright WM. (1973). Faciolingual tooth movement: its influence on the root and cortical plate.. Am J Orthod..

[25] Bimstein E, Crevoisier RA, King DL (1990). Changes in the morphology of the buccal alveolar bone of protruded mandibular permanent incisors secondary to orthodontic alignment. Am J Orthod Dentofacial Orthop.

[26] Patcas R, Müller L, Ullrich O, Peltomäki T (2012). Accuracy of cone-beam computed tomography at different resolutions assessed on the bony covering of the mandibular anterior teeth. Am J Orthod Dentofacial Orthop.

[B27] Ruf S, Hansen K, Pancherz H (1998). Does orthodontic proclination of lower incisors in children and adolescents cause gingival recession?. Am J Orthod Dentofacial Orthop.

[28] Aziz T, Flores-Mir C (2011). A systematic review of the association between appliance-induced labial movement of mandibular incisors and gingival recession. Aust Orthod J.

[29] Helm S, Petersen PE (1989). Causal relation between malocclusion and periodontal health. Acta Odontol Scand.

[30] Sun Z, Smith T, Kortam S, Kim DG, Tee BC, Fields H (2011). Effect of bone thickness on alveolar bone-height measurements from cone-beam computed tomography images. Am J Orthod Dentofacial Orthop.

